# Inhibition of protein PMP22 enhances etoposide-induced cell apoptosis by p53 signaling pathway in Gastric Cancer

**DOI:** 10.7150/ijbs.59825

**Published:** 2021-07-25

**Authors:** Jingjing Hou, Lin Wang, Jiabao Zhao, Huiqin Zhuo, Jia Cheng, Xin Chen, Wei Zheng, Zhijun Hong, Jianchun Cai

**Affiliations:** 1 Department of Gastrointestinal Surgery, Zhongshan Hospital of Xiamen University, Xiamen, Fujian 361004, China; 2 Institute of Gastrointestinal Oncology, Medical college of Xiamen University, Xiamen, Fujian 361004, China; 3 Xiamen Municipal Key Laboratory of Gastrointestinal Oncology, Xiamen 361004, Fujian, China

**Keywords:** PMP22, etoposide, p53, Apoptosis, gastric cancer

## Abstract

Gastric Cancer (GC) is one of the main causes leading to death. PMP22, as a member of the GAS3 family of tetraspan proteins, it is associated with a variety of neurological diseases. Recently, more and more studies have shown that PMP22 play a great role in the physiological processes such as cells adhesion, migration, proliferation and tumorigenesis, but the involvement and functional mechanisms of PMP22 in Gastric carcinoma are not investigated clearly. In this study, we found that the PMP22 was overexpressed in the GC cells and tissue. Knockdown of PMP22 inhibits cell growth. Over-expressed PMP22 inhibits the etoposide-induced apoptosis, meanwhile knockdown of PMP22 promotes the etoposide-induced proliferation suppression, and increases cell apoptosis in GC cells. Furthermore, PMP22 enhanced the inhibition of the p53 transcriptional activities and down-regulated the p53 targeting genes, including p21, BAX and PUMA with or without treatment of etoposide. Finally, our results showed that PMP22 reduced the etoposide-induced tumor growth suppression in nude mice. Taken together, our research provided an anti-apoptotic properties alternative mechanism for PMP22 in gastric carcinoma and suggested PMP22 can be a potential target for the treatment of gastric cancer.

## Introduction

The incidence and mortality of gastric cancer (GC) is the fifth most common digestive system cancer worldwide 1 and is the third highest cause of cancer-related deaths in China 2-5. Interactions between genetic and environmental elements promote aberrant activation of oncogenic signaling pathways that can accelerate cellular transformation and tumourigenesis^2^. Although there have been significant advances in GC treatment, the 5-year survival rate of GC remains less than 25 percent 2, 4. Given this, the molecular mechanism of GC should be investigated to improve clinical treatment, develop early diagnostic markers, and obtain new therapeutic options for GC patients 4, 6. However, the underlying molecular mechanisms of GC are largely unknown.

Apoptosis is one of the main types of programmed cell death (PCD) and plays important roles in cell and tissue homeostasis and in growth control 7, 8. Consequently, the deregulation of apoptosis is commonly associated with several kinds of diseases, including cancers 8, 9. P53 is an important protein that is involved in the process of apoptosis. As a transcription factor, p53 is mutated in most human cancers 10-12. Oncogenes, DNA damage, ionizing radiation, and chemotherapeutic agents including cisplatin and etoposide can increase p53 protein levels 13, 14. To suppress cancer, p53 protein regulates the transcription of many different genes, including BAX, Puma, NOXA1, CDKN1A, and GADD45A, in response to a wide variety of stress signals including DNA repair, cell-cycle arrest, senescence, and apoptosis 9, 11, 13. Mutations in tumors can make them resistant to apoptosis induction through the p53 pathway, therefore targeting apoptosis pathways is an attractive strategy for cancer therapy^8^.

Peripheral myelin protein 22 (PMP22) is a 22-kDa tetraspan glycoprotein, which is expected to predominantly expressed by myelinating Schwann cells and is closely related to Charcot-Marie-Tooth disease (CMT)^15^, ^16^. Several recent studies have shown that PM22 participates in cell proliferation and tumorigenesis in various cancers. However, the function of PM22 in tumors remains unclear. Several studies have indicated that PMP22 is a potential tumor suppressor 17-22, but other studies report a potential oncogenic function of PMP22 23-31. Overall, the involvement and functional mechanisms of PMP22 in gastric carcinoma are poorly understood.

To comprehensively study the function of PMP22 in apoptosis during gastric carcinogenesis, we examined PMP22 expression in GC tissue and a GC cell line. We found that PMP22 was overexpressed in GC tissue and knockdown of PMP22 inhibited cell proliferation. When we treated cells with the chemotherapy drug etoposide, PMP22 inhibited etoposide-induced apoptosis, and the knockdown of PMP22 promoted etoposide-induced suppression of proliferation and increased apoptosis in GC cells. Over-expressed PMP22 also inhibited p53 transcriptional activities and down-regulated expression of p53-targeting genes, including p21, BAX, and PUMA in the presence of etoposide. Our results revealed that PMP22 inhibits etoposide-induced cell apoptosis via activating the p53 signaling pathway in GC. These findings suggest that PMP22 exhibits anti-apoptotic properties in gastric carcinoma, and raise the possibility of PMP22 as a potential target for the treatment of gastric cancer.

## Material & Methods

### Plasmids construction

Full-length cDNA encoding human PMP22 was amplified by PCR, the PCR product was sub-cloned into pLV-CMV, pCMV-HA vectors to get PMP22 overexpressing plasmids. Luciferase reporter plasmids PGL-3-p53 RE-luc, PGL-3-p21-luc and PGL-3-Bax-luc were kindly provided by professor Jiahuai Han (Xiamen University, Xiamen, China). All constructs derived from PCR products were verified by DNA sequencing.

### RNA interference

The pLV lentiviral vector was used to express short hairpin RNA directed against the PMP22 or LacZ control sequence (GTCTCCGAACGTGTCACGTT). Oligonucleotides targeting PMP22 (PMP22 shRNA-1, 5'- CCAAACTCAAACCAAACCAAA -3'; PMP22 shRNA-2, 5'- CGGTGTCATCTATGTGATCTT -3') were cloned into the pLKO.1 lentiviral vector. Recombinant lentiviral plasmids were cotransfected into 293T cells with the packaging plasmids VSV-G, RSV-REV, and pMDL. After 48 h the viral supernatants were passed through 0.45-μm filters and used to infect target cells in the presence of 8μg/ml polybrene (Sigma-Aldrich).

### Cell culture, transfection and treatment

The HGC27, SGC7901 and other human gastric cancer cell lines (GES, MKN28, AGS, MKN45, BGC823, MGC803) were purchased from the Institute of Cell Biology (Shanghai, China, http://www.cellbank.org.cn) and cultured in RPMI-1640 (Gibco, Life Technologies, NY, USA) supplemented with 10% fetal bovine serum (FBS, Excell), 100 U/ml penicillin and 100µg/ml streptomycin (Gibco). Plasmid DNA transfection were performed with Turbofect reagent (Invitrogen, Carlsbad, CA, USA) according to the manufacturer's instructions. Etoposide was purchased from Sigma and was added to subconfluent cells at the indicated doses.

### Real-time quantitative PCR (qPCR)

For qPCR analyses of mRNA, reverse transcription was performed with TRIzol (Invitrogen, Carlsbad, CA, USA) extracted total RNAs using a ReverTra Ace-α® Kit as instructed (Toyobo, Tokyo, Japan). qPCR was performed using the SYBR Green Real-Time PCR Master Mix (Toyobo) and the Step One Plus Real-Time PCR system (Applied Biosystems Inc., Foster City, CA, USA) according to the manufacturers' protocols with primers showed in Table 1.

### Clinical samples

All clinical samples were collected with the informed consent of the patients and study protocols that were in accordance with the ethical guidelines of the Declaration of Helsinki (1975) and were approved by the Institutional Medical Ethics Committee of Xiamen University. GC pathological diagnosis was verified by at least two pathologists. 40 human GC specimens and paired adjacent epithelial tissues were obtained from the Shanghai OUTDO BIOTECH CO., LTD.

### Immunohistochemistry (IHC) analysis

After deparaffinization, rehydration and antigen-retrieval, hepatic tissue slides (4-7 μm) were blocked by 3% H_2_O_2_ for 10 min and incubated with anti-PMP2 antibody (Abcam, Cambridge, MA, USA) at 4 °C overnight. The slides were then stained with horseradish peroxidase (HRP)-labeled IgG (Shanghai Long Island Biotec, Shanghai, China) at 25 °C. Subsequently, the sections were stained with diaminobenzidine (DAB), counterstained with hematoxylin and washed in water. The immunoreactive cells were counted in five visual fields of each section under a 200×light microscope.

### Cell counting kit-8 (CCK-8) assay

Relative cell viability of gastric cells treated with etoposide was detected with a CCK-8 kit (Dojindo, Kumamoto, Japan). Briefly, cells were plated into 96-well plates containing 100 μl of growth medium, 48 h later, CCK-8 reagents (10μL /well) were added and incubated for 3 h at 37°C in a 5% CO_2_ incubator. The absorbance was measured at 490 nm. The cell viability was calculated as follows: relative cell viability %=[(A_1_-A_B_)/(A_0_-A_B_)] ×100%, A_1_ is the absorbance of treatment group, A_0_ is the absorbance of control group and A_B_ is the absorbance of blank group.

### Colony formation assay

Gastric cancer cells were first infected with pLV-Ctrl or pLV-PMP22 lentivirus or infected with pLKO-shCtrl, pLKO-shPMP22-1 or pLKO-shPMP22-2 lentivirus respectively, then 1×10^3^ cells/well were seeded in 6-well plates with medium changed every two days, Cells were fixed with methanol and stained with violet after 10 days. Colonies were counted and analyzed for clonogenicity.

### Luciferase reporter assay

SGC7901 cells or HGC27cells were transfected in 6-well dishes at 80% confluence with 0.5 μg different reporters, together with other plasmids in different combinations as indicated. Each sample was supplemented with 0.5 μg of pCMV5-LacZ, which expresses β-galactosidase, for monitoring the transfection efficiency. The cells were collected, and the luciferase activity was measured at 24 h after transfection. All transfections experiments were performed at least five times in triplicate, and the error bars represent SD of the means.

### Western blots

Cells or tissues were lysed in a lysis buffer and protein concentrations for cells or tissues lysates were measured using the BCA protein assay kit (Thermo Fisher Scientific Inc., Waltham, MA, U.S) or G250 (Sigma-Aldrich, St. Louis, MO, USA). Thirty micrograms protein/lane whole cell lysates were electrophoresed in SDS-PAGE and transferred to a PVDF membrane (Millipore, Billerica, MA, USA). After blocking for 1 h at room temperature in TBST with 5% non-fat milk, the membranes were probed with the following primary antibodies: PMP22 (1:500 CST), actin (1:5000 Sigma). After washing three times, the membranes were incubated with HRP-conjugated goat anti-mouse or anti-rabbit secondary antibodies, 1:5000 (BD). Then, the chemiluminescence reaction was performed.

### Flow cytometry

Apoptosis was measured using Annexin V-FITC/PI (Ebioscience, San Diego, USA) dual staining by flow cytometry. Briefly, SGC7901 cells (2×10^ 5^ /well) were seeded into 6-well plates and exposed to etoposide for 24 hours. Cells were harvested and washed in cold FACS buffer (PBS containing 2% FBS), and labeled with Annexin V-FITC for 30 min at 4°C in the dark and then with PI. The stained cells were analyzed by flow cytometry (LSRFortessa, Becton Dickinson, San Jose, CA, USA).

### Tumor xenografts

Four to six-week-old male nude mice were obtained from the Laboratory Animal Center of Xiamen University. The animals were maintained on standard laboratory chow under a 12 h/12 h light/dark schedule, unless otherwise indicated. All animal experiments were conducted according to protocols and guidelines approved by the Xiamen University Institutional Animal Care and Use Committee. A total of 6×10^6^ control and SGC7901-PMP22 cells were subcutaneously injected into the dorsal flanks of nude mice, respectively. From day 10 after the injection of cells, the sizes of the tumors were measured every 4 days using a vernier caliper along two perpendicular axes. The volumes of the tumor were calculated using the formula: Volume=Length×Width 2×0.52.

### TCGA analysis

We used mRNA expression array datasets from TCGA to explore gene expression profiles in human cancer. We downloaded data from 375 tumor tissues and 32 normal tissues of mRNA expression data to determine differences in transcription levels of FAK between normal gastric tissues and GC tissues. The data regarding mRNA expression were produced on the platforms of Illumina Infinium HumanMethylation450 BeadChip and IlluminaGA_RNASeqV2.1.0.0 (Illumina, Inc., San Diego, CA, USA).

### Survival analysis

Overall survival (OS) curve was calculated with the Kaplan-Meier method to evaluate the prognostic value of PMP22 mRNA expression in GC (Gastric Cancer). A total of 876 GC patients were recruited from the Kaplan-Meier Plotter online database. Subjects were split into two groups by median expression (high *vs*. low expression) and assessed by a Kaplan-Meier survival plots.

### Statistical analysis

Values represent the mean ± SD for at least three independent experiments. One-way ANOVA with Bonferroni's post-test was used for multiple comparisons and the Student's t test (two-tailed) was used for pair-wise comparisons. Correlation analyses were performed with Pearson's test. *P* values < 0.05 were considered statistically significant.

## Results

### PMP22 is significantly upregulated in human gastric cancer cell lines and clinical samples

**Figure 1:**
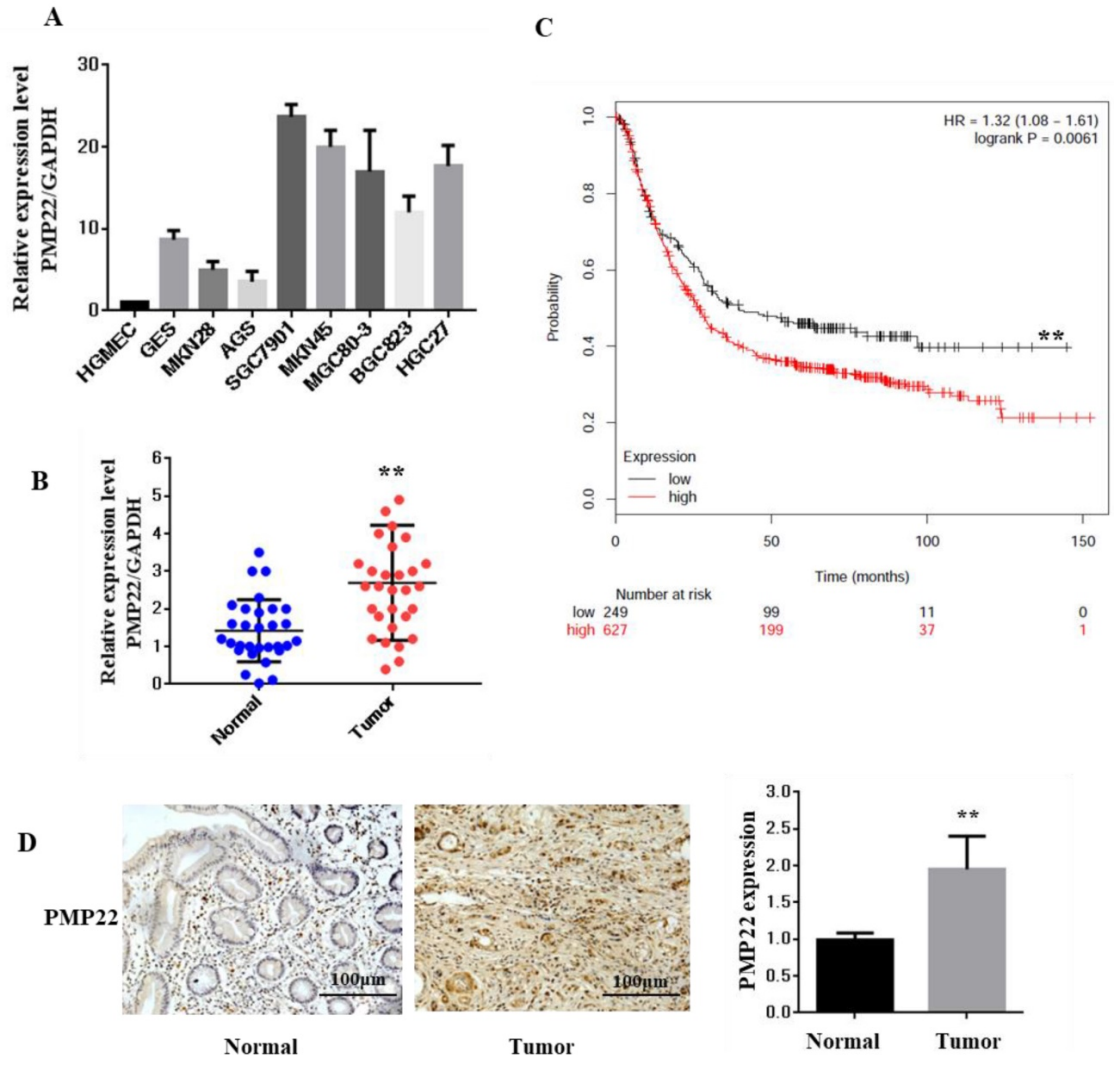
Expression of PMP22 in Gastric cancer (GC) tissues and cell lines. (A) The expression of PMP22 was detected using qRT-PCR in different GC cell lines (HGMEC, GES, MKN28, AGS, SGC7901, MKN45, BGC823, MGC803, and HGC27). (B) Kaplan-Meier survival curves for GC patients were plotted based on high or low PMP22 expression level. (C) The expression level of PMP22 was measured in 40 pairs of GC tissues and adjacent normal tissues by qualitative real-time reverse transcriptase PCR (qRT-PCR). Tumor, Gastric cancer tissue; Normal, adjacent noncancerous colon tissue; GAPDH mRNA were calibrated for qPCR analysis. (D) Immunohistochemistry results of PMP22 in human GC tissues. Results are representative of three independent experiments, and the error bars represent the SD. *p < 0.05; ** p < 0.01; *** p < 0.001.

**Figure 2:**
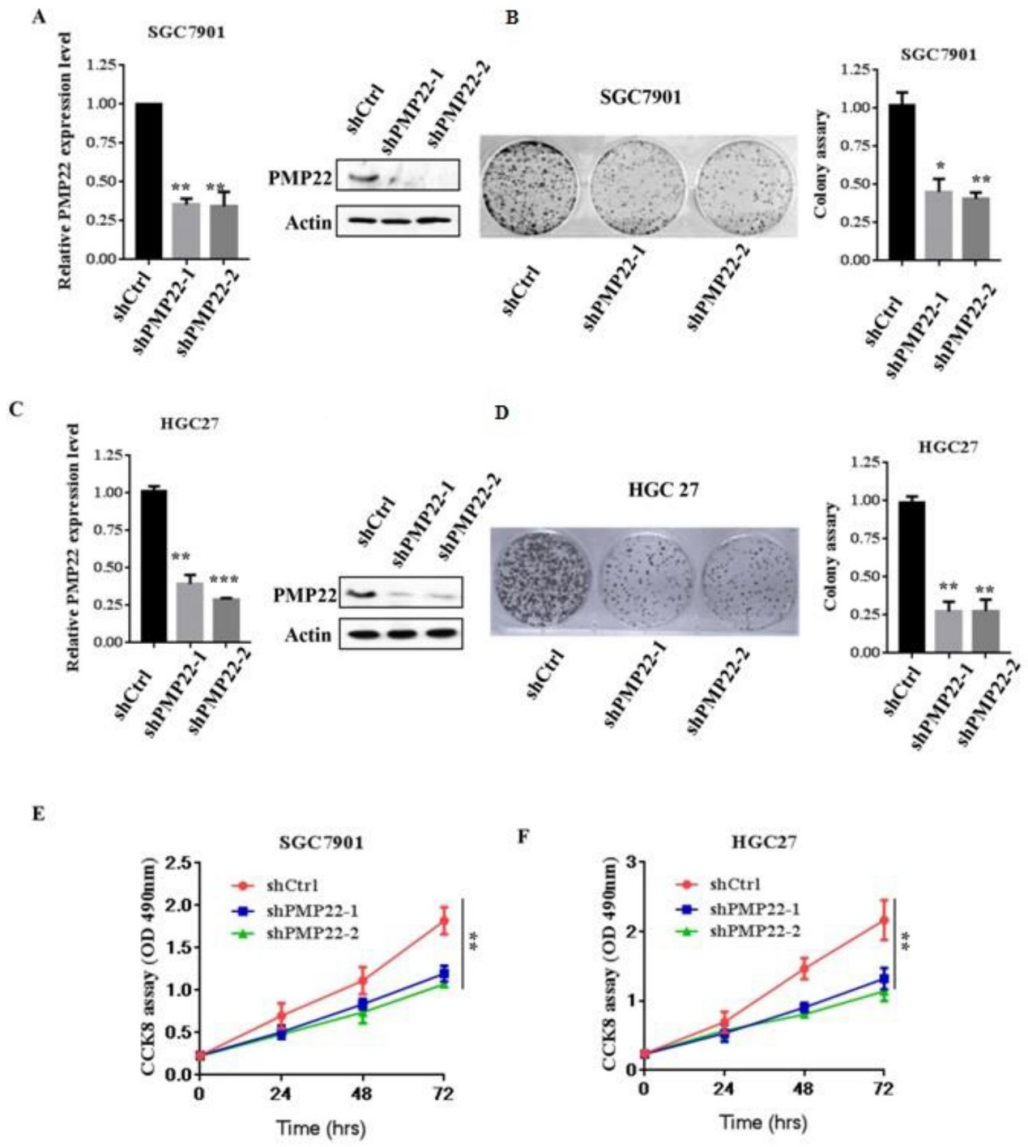
Knockdown of PMP22 inhibits cell growth and GC cell proliferation *in vitro*. (A) SGC7901 cells were infected with lentivirus expressing either pLKO-shPMP22 or pLKO-shCtrl for 72 hours and then the mRNA and protein expression levels of PMP22 were examined by q-PCR and Western blot. (B) Colony formation assay of SGC7901-shCtrl cells SGC7901-shPMP22 cells. (C) HGC27 cells were infected with lentivirus expressing either pLKO-shPMP22 or pLKO-shCtrl for 72 hours and the mRNA and protein expression levels of PMP22 were examined by q-PCR and Westerin blot. (D) Colony formation assay of HGC27-shCtrl cells and HGC27-shPMP22 cells. (E) Cell viability was determined in SGC7901-shCtrl cells and SGC7901-shPMP22 cells by CCK-8 assay. (F) Cell viability was determined in HGC27-shCtrl cells and HGC27-shPMP22 cells by CCK-8 assay. Statistical analysis of the CCK-8 assay results at 72h shown in panel E. Results are representative of three independent experiments, and the error bars represent the SD. *p < 0.05; ** p < 0.01; *** p < 0.001.

PMP22 is well known as an integral membrane glycoprotein of the peripheral nervous system. To explore the function and relationship between the expression of PMP22 and gastric carcinogenesis, we first examined the expression of PMP22 in different gastric cell lines. As shown in Fig. 1A, compared with the expression level in a human glomerular microvascular endothelial cell line (HGMEC), PMP22 was upregulated in most of the gastric cancer cell lines. Next, the relative expression levels of PMP22 in the GC tissue group and the corresponding pathologically noncancerous gastric tissue group (control group) were evaluated by RT-PCR assay. PMP22 was significantly upregulated in comparison with normal tissues (Fig. 1C). Further analysis in Table 1 showed that PMP22 level was correlated to tumor-node-metastasis TNM staging (n = 40, p < 0.05), while no apparent association was found between PMP22 expression with patient gender, patient age, tumor size. The detailed description of the patient information is shown in Table 2 (Age, Tumor size, Clinical staging, etc.). As shown in Fig. 1B, overall survival (OS) curves were plotted using the Kaplan-Meier method based on the gene expression levels in 876 GC samples. Patients with higher levels of PMP22 had significantly shorter OS (Fig. 2A, logrank p = 0.0061) than those with lower levels of PMP22. Immunohistochemical analysis also showed that significant expression of PMP22 in 29/48 (60%) of GC tissues, with expression levels that were higher in GC than that in adjacent noncancerous gastric tissues (Fig. 1D). These results suggested that PMP22 may play an important role in GC development.

### Downregulation of PMP22 suppressed gastric cancer cell proliferation

To evaluate the effects of PMP22 on gastric carcinogenesis, we used a lentivirus system to knockdown of PMP22 in SGC7901 and HGC-27 cells (High expression of PMP22). SGC7901 and HGC-27 gastric cancer cells were infected with lentivirus expressing either pLKO-shCtrl or pLKO-shPMP22 for 72 hours, and then the mRNA expression levels of PMP22 were then determined by RT-PCR assay. As shown in Fig. 2A and Fig. 2C, compared to the control shRNA group, mRNA expression after PMP22 knockdown was reduced by 65% and 75%, respectively in the SGC7901 cells (*p* < 0.001,* p* < 0.01) and 70% in the HGC-27 cells (*p* < 0.001,* p* < 0.01). The colony formation assay was next applied to examine the relative cell proliferation. As shown in Fig. 2B and Fig. 2D, proliferation was suppressed after downregulation of PMP22 by 70% (SGC7901) and 75% (HGC27) compared with the control group. Consistently, the CCK-8 assay results also showed reduced proliferation of shPMP22 cells compared with that of shCtrl cells (*p* < 0.05, *vs* control group) (Fig. 2E and Fig. 2F). Taken together, these results demonstrated that the inhibition of PMP22 significantly suppressed gastric cancer cell proliferation.

### PMP22 inhibits etoposide-induced cell apoptosis in gastric cancer cells

**Figure 3:**
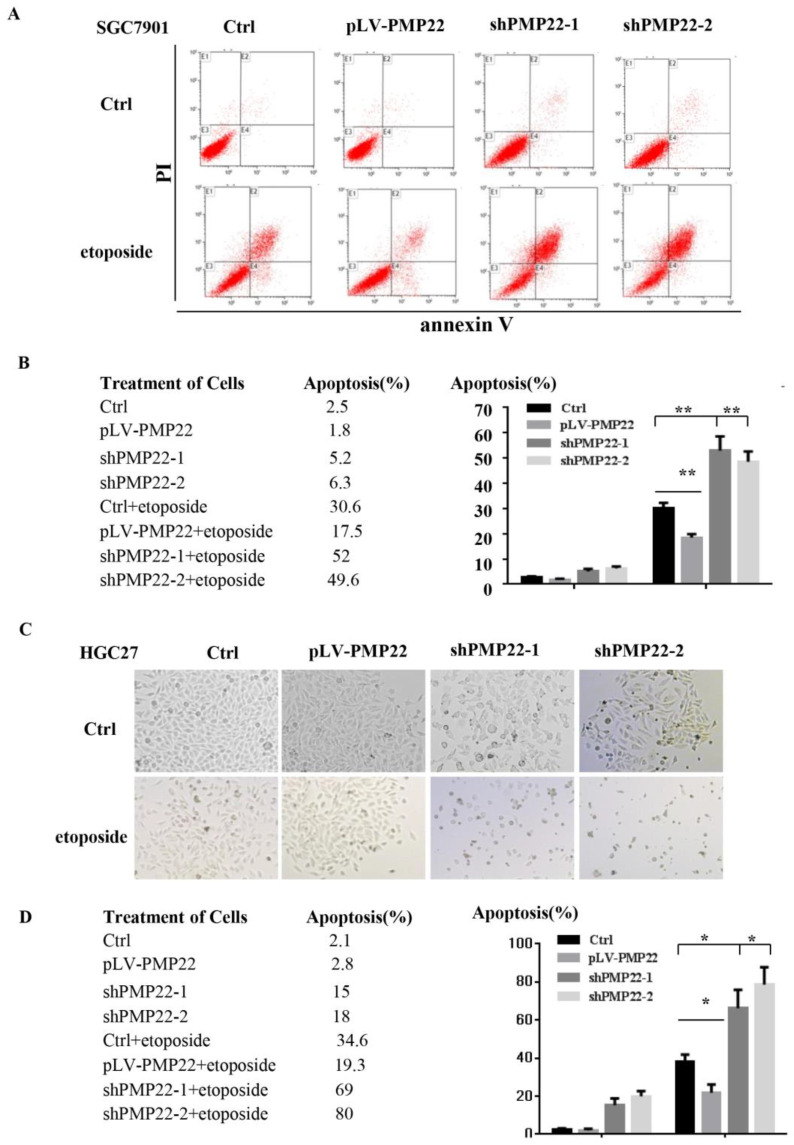
PMP22 inhibits etoposide-induced cell apoptosis. (A) Flow cytometry to assess cell apoptosis. After overexpression of PMP22 or knockdown of PMP22 in the SGC7901, cells were treated with PBS or etoposide for 12h. Cells were collected and apoptosis was examined by flow cytometry assay. (B) Statistical analysis of data presented in panel A. The apoptosis rates are shown. Results are representative of three independent experiments, and the error bars represent the SD. *p < 0.05; ** p < 0.01; *** p < 0.001. (C). PMP22-overexpressing HGC27 cells (HGC27-PLV-PMP22) showed a decreased apoptotic morphology after etoposide treatment, and HGC27-shPMP22 cells showed an increased apoptotic morphology after etoposide treatment. The indicated cells were plated in 12-well plates. The next day, the cells were treated with etoposide for 12 h, and the cells were imaged with a Nikon-TE2000 microscope. (D) Statistical analysis of images in panel C. *p < 0.05; **p < 0.01; ***p < 0.001.

**Figure 4:**
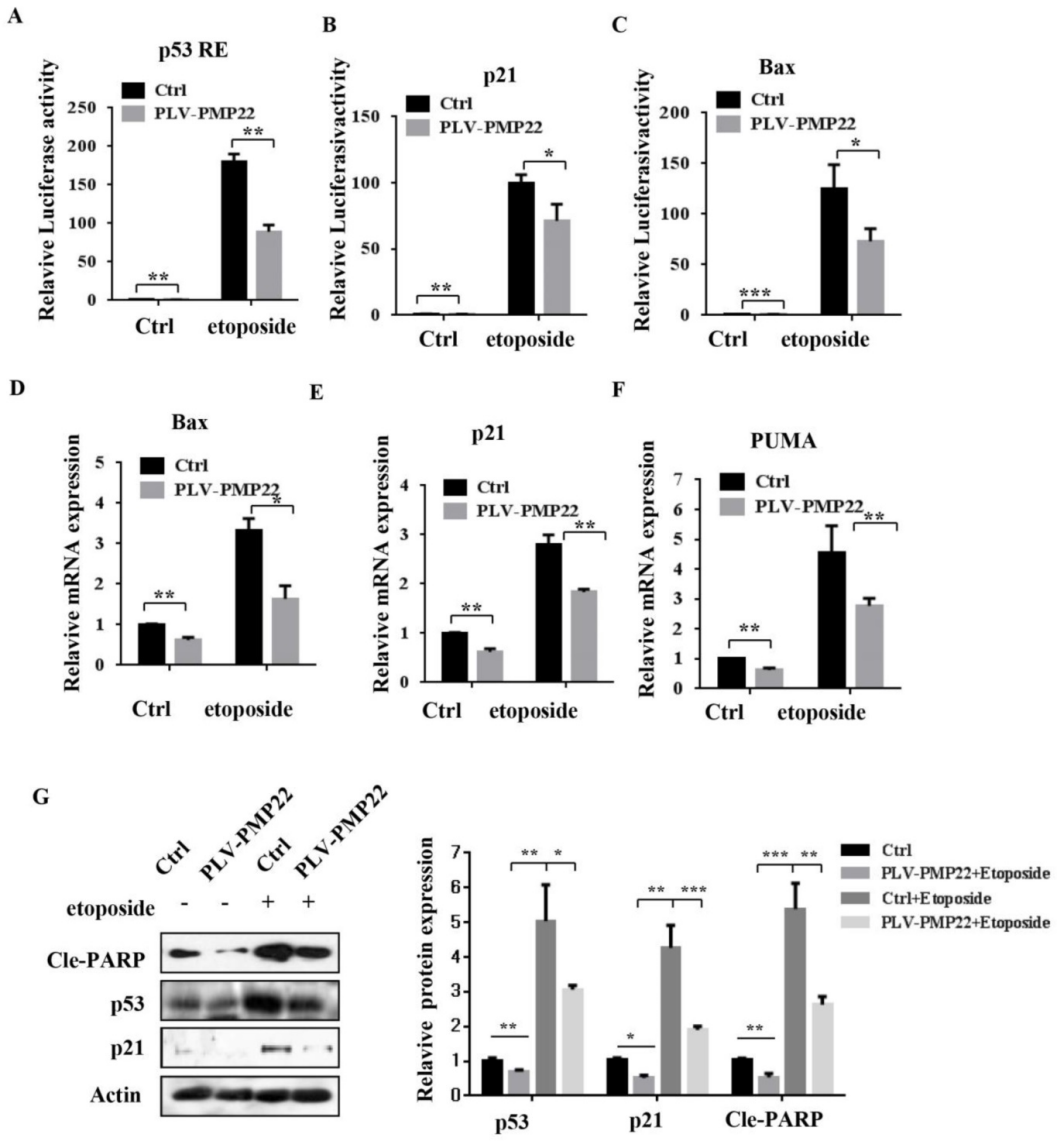
Overexpression of PMP22 represses the etoposide-induced activation of p53 target genes. (A-C) Luciferase reporter assays of p53RE, p21, and Bax. SGC7901-PLV-Ctrl or SGC7901-PLV-PMP22 cells were treated with or without etoposide, and then luciferase reporter assays were performed. (D-E) Analysis of Bax, p21, and PUMA mRNA expression by q-PCR. SGC7901-PLV-Ctrl or GC7901-PLV-PMP22 cells were treated with or without etoposide, and then assayed. (F) SGC7901-PLV-Ctrl or SGC7901-PLV-PMP22 cells were treated with or without etoposide, and then the expression of apoptosis-associated proteins were detected by western blots with anti-p53, p21, Cle-PARP and Actin antibodies, and the expression of protein were quantified and statistically analyzed using image analyzer. (G) Statistical analysis of the protein expression showed in (F). Results are representative of three independent experiments, and the error bars represent the SD. *P < 0.05; **P < 0.01; ***P < 0.001.

Apoptosis plays a critical role in development and homeostasis 32, 33. To test whether PMP22 is related to the regulation of apoptosis, we used etoposide, a chemotherapeutic drug, to induce apoptosis. Annexin V-FITC assay was performed to investigate the effect of PMP22 on etoposide-induced apoptosis. The results in the Fig.3A illustrated that etoposide treatment alone resulted in a 30.6% apoptotic rate, however, the percentage of apoptotic cells after etoposide treatment decreased to 17.5% upon overexpression of PMP22, while the percentage of apoptotic cells increased to about 50% after PMP22 silencing. Fig. 4B shows the corresponding values. The same result was also observed in another gastric cancer cell, HGC-27 (Fig. 3C-D). Compared to control cells, PMP22-overexpressing HGC27 cell lines presented reduced apoptotic cell death and the PMP22-knockdown HGC27 cell lines presented increased apoptotic cell death after etoposide treatment (Fig. 3C-D). These results suggested that PMP22 inhibited apoptosis in gastric cancer cells.

### PMP22 inhibits etoposide-induced cell apoptosis by inhibiting the transcriptional activity of p53

Apoptosis is closely related to function of the p53 signaling pathway and our previous results showed that etoposide-induced N-Myc interacting protein (NMI) inhibited proliferation and promoted apoptosis by activating p53 signaling. To examine whether PMP22 affected the p53 signaling pathway and transcriptional activities of p53, we performed luciferase reporter assays in SGC7901 cells. The results showed that etoposide treatment enhanced activation of several p53-target genes, including p53 RE, p21, and Bax, and overexpression of PMP22 significantly decreased the extent of activation by etoposide treatment (Fig. 4A-C). We also characterized the mRNA levels of p53-target genes, Bax, p21, and PUMA after overexpression of PMP22. The results showed that PMP22 overexpression decreased p53 transcriptional activities compared with the control groups with and without etoposide treatment (Fig. 4D-F), The western blots results showed that PMP22 inhibits etoposide-induced transcriptional activity of p53, and inhibits the cleavage of PARP (Fig. 4G).

**Figure 5:**
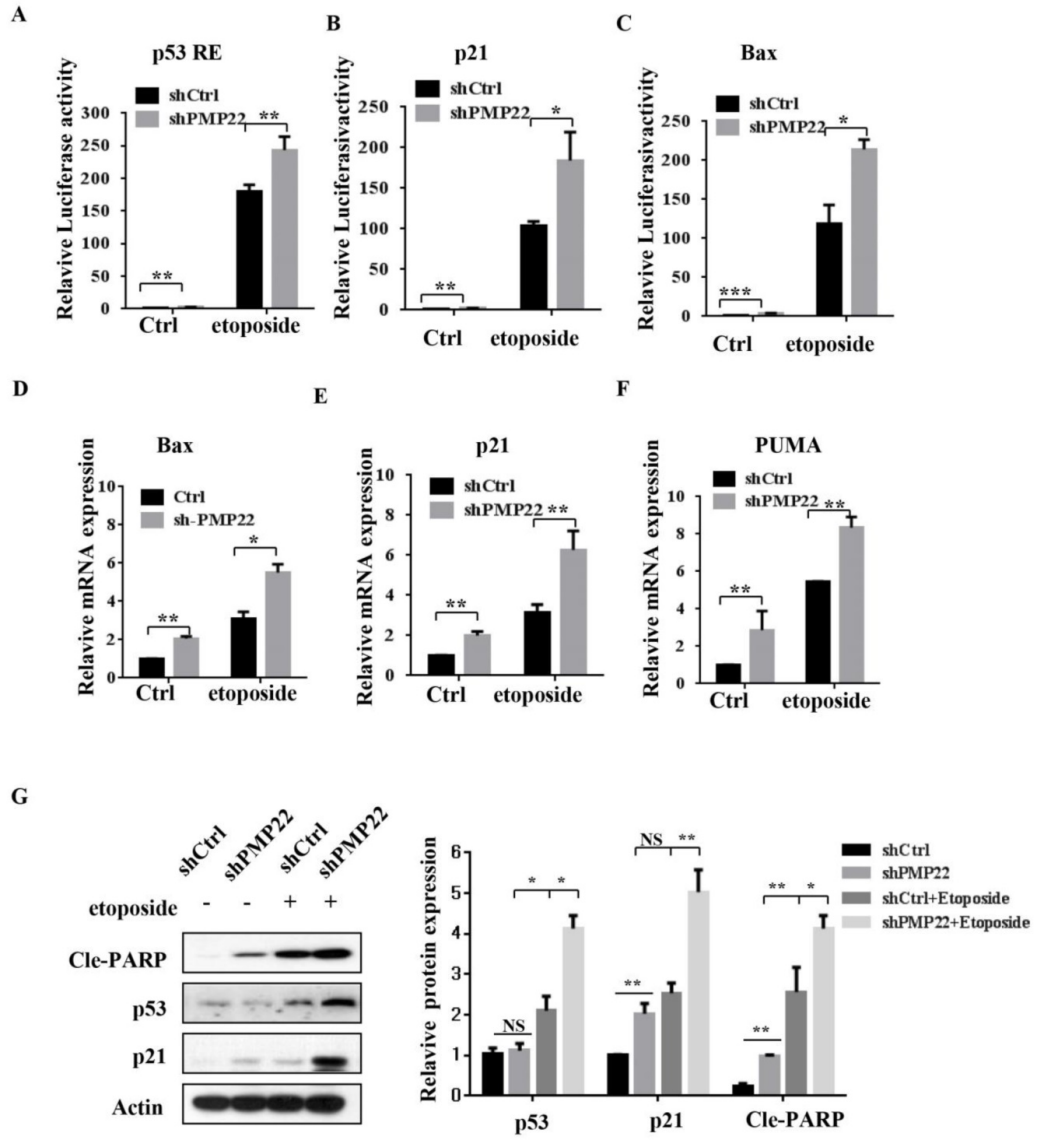
Knockdown of PMP22 increases the etoposide-induced activation of P53 target genes. (A-C) Luciferase reporter assays of p53RE, p21, and Bax. SGC7901-PLKO-shCtrl or SGC7901-PLKO-shPMP22 cells were treated with or without etoposide, and then luciferase reporter assays were performed. (D-E) Analysis of Bax, p21, and PUMA mRNA expression by q-PCR. SGC7901-PLKO-shCtrl or SGC7901-PLV-shPMP22 cells were treated with or without etoposide before qPCR. (F) SGC7901-PLKO-shCtrl or GC7901-PLKO-shPMP22 cells were treated with or without etoposide, and then the expression of apoptosis-associated proteins were detected by western blots with the indicated antibodies and quantified and statistically analyzed using image analyzer. (G) Statistical analysis of the protein expression showed in (F). Results are representatives of three independent experiments, and the error bars represent the SD. *P < 0.05; **P < 0.01; ***P < 0.001.

**Figure 6:**
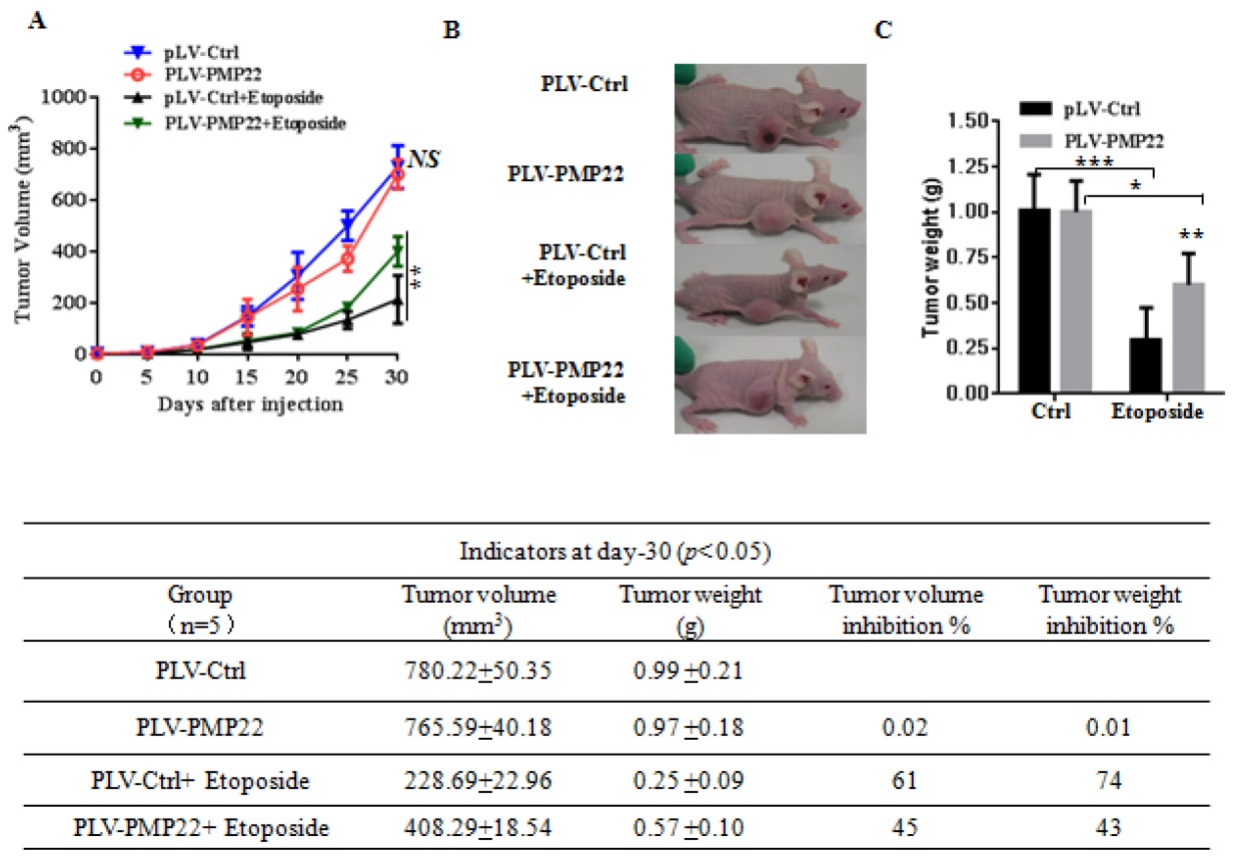
PMP22 reduces etoposide-induced tumor growth inhibition. (A) The tumor growth curve. SGC7901-Ctrl and SGC7901-PMP22 cells were inoculated into nude BALB/c mice. After tumors reached 50 to 100 mm3 in size (15 days after implantation), mice were treated with PBS or etoposide every 3 days for 15 days. Tumor formation was monitored, and the tumor sizes were measured every 4 days. (B) Photographs of the xenograft from various groups of nude mice treated as indicated. (C) Statistical analysis of the tumor weight of each group. *p < 0.05; **p < 0.01. (D) Statistical analysis of the tumor volume inhibition rate and tumor weight inhibition rate (n=5). *p < 0.05; **p < 0.01; ***p < 0.001.

We also performed luciferase reporter assays in SGC7901 cells with lentivirus expressing either pLKO-shCtrl or pLKO-shPMP22. In contrast to the result shown in Fig. 5A-C, knockdown of PMP22 increased the transcriptional activities of p53 RE, p21, and Bax (Fig. 6A-C), and RT-PCR assay also showed that knockdown of PMP22 significantly enhanced mRNA expression of these genes upon etoposide treatment (Fig. 5D-F). We also examined the protein expression of p53, p53 target gene p21, and Cleavage-PARP, the results showed that knockdown of PMP22 increases etoposide-induced transcriptional activity of p53, and increases the cleavage of PARP (Fig. 5G).

### PMP22 inhibits etoposide-induced tumor growth suppression and knockdown of PMP22 enhances etoposide-induced tumor growth suppression in nude mice

**Figure 7:**
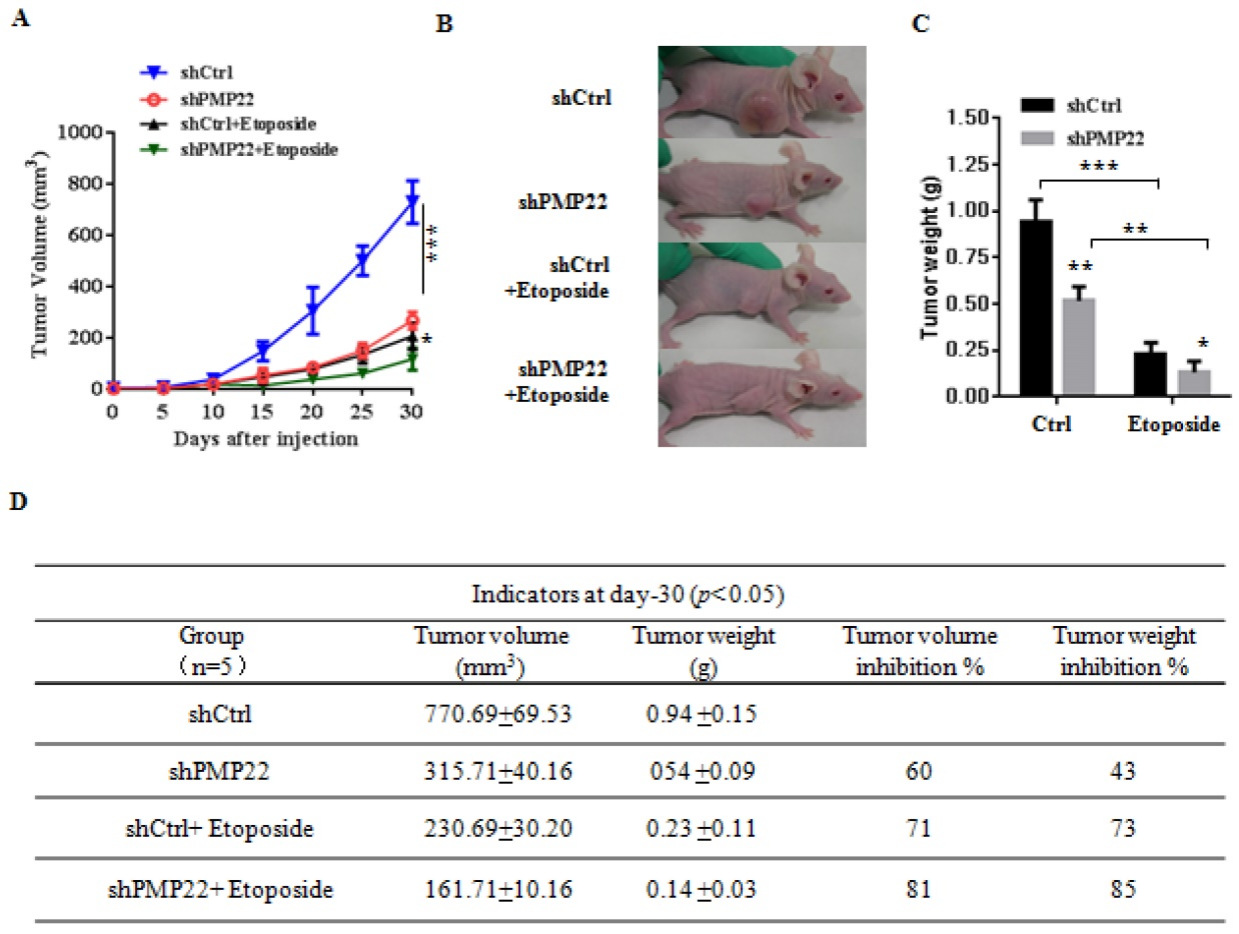
Knockdown of PMP22 increases etoposide-induced tumor growth inhibition. (A) The tumor growth curve. SGC7901-shCtrl and SGC7901-shPMP22 cells were inoculated into nude BALB/c mice. After tumors reached 50 to 100 mm3 in size (15 days after implantation), Mice were treated with PBS or etoposide for 15 days. Tumor formation was monitored, and tumor sizes were measured every four days. (B) Photographs of the xenograft from various groups of nude mice treated as indicated. (C) Statistical analysis of the tumor weight of each group. *p < 0.05, **p < 0.01. (D) Statistical analysis of the tumor volume inhibition rate and tumor weight inhibition rate (n=5). *p < 0.05; **p < 0.01; ***p < 0.001.

To examine the effects of PMP22 in gastric cancer development *in vivo*, xenograft tumors were induced in nude mice by a single injection of SGC7901-Ctrl and SGC7901-PMP22 cells at a dosage of 5×10^6^. After tumors reached 50 to 100 mm^3^ in size (15 days after implantation), mice were treated with PBS or etoposide every 3 days for 15 days. Tumor formation was monitored, and the tumor sizes were measured every 4 days. Thirty days after implantation, we observed that the sizes of xenograft tumors in mice injected with SGC7901-PMP22 cells were larger than the tumors in mice injected with SGC7901-Ctrl cells after treatment of etoposide, while the difference between the two groups treated with PBS was not very prominent (Fig. 6A-B) Consistent with this result, knockdown of PMP22 reduced tumor growth, and this inhibition was more obvious after etoposide treatment (Fig. 7A-B). As shown in Fig.7C, when treated with etoposide, the PMP22 overexpression group showed an increased tumor weight compared to the Ctrl group (Fig. 6C). Additionally, we observed that knockdown of PMP22 reduced tumor weight, and this inhibition was more obvious after etoposide treatment (Fig. 7C). The volume inhibition rate and tumor weight inhibition rate were statistically analyzed, as shown in Fig.6D and 7D. These data showed that the volume inhibition rate and tumor weight inhibition rate of the group that received from overexpressed PMP22 and etoposide treatment were both lower compared than those of the Ctrl group with etoposide. The knockdown of PMP22 increased both the volume inhibition rate and the tumor weight inhibition rate. Overall, our results demonstrated that PMP22 overexpression enhanced the tumorigenicity of gastric cancer cells and also inhibited etoposide-induced tumor suppression.

## Discussion

**Figure 8:**
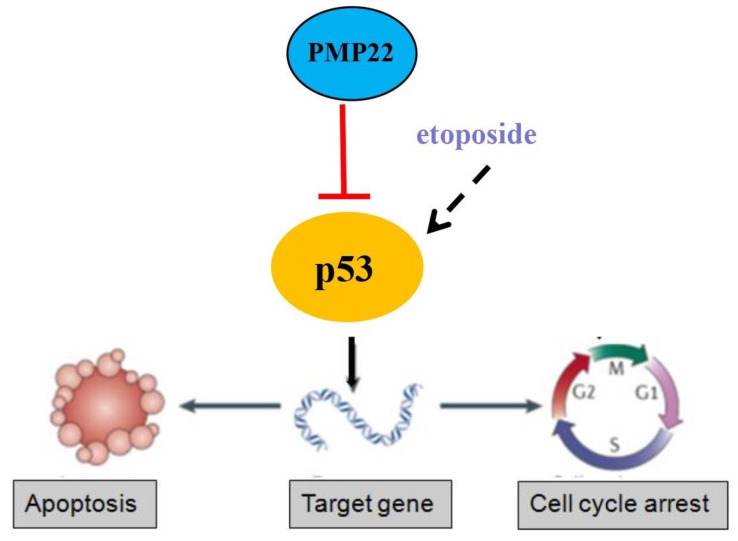
Proposed working model of PMP22 in the regulation of gastric cancer. PMP22 inhibits etoposide induced-cell apoptosis via p53 signaling pathway in gastric cancer.

Gastric carcinoma (GC) is a deadly malignancy afflicting about one million people worldwide, and the poor diagnosis and lack of effective therapies result in a low survival rate 1, 3, 4, 34, 35. Although many studies have reported the diagnosis and treatment of gastric cancer, the underlying molecular mechanisms in GC development and progression remain unclear 3, 36, 37. Here, we show that PMP22 regulates gastric cancer cell proliferation by inhibiting cell apoptosis. Our results showed that: (1) PMP22 is significantly upregulated in human gastric cancer cell lines and clinical samples (Fig. 1); (2) Downregulation of PMP22 suppressed gastric cancer cell proliferation (Fig. 2); (3) PMP22 inhibits etoposide-induced cell apoptosis in gastric cancer cells (Fig. 3); (4) PMP22 suppressed p53 transcriptional activity upon etoposide treatment; (Fig. 4-5); (5) PMP22 enhanced tumorigenicity *in vivo* in nude mice and inhibited etoposide-induced tumor growth inhibition (Fig. 6-7). A possible pattern diagram was constructed, illustrating the potential molecular mechanism by which PMP22 regulates cell proliferation (Fig. 8). Collectively, our results demonstrate a novel anti-apoptotic role of PMP22 in the progression of gastric cancer, suggesting that PMP22 might be an important diagnostic or therapeutic target for gastric cancers and other human diseases.

PMP22 is a tetraspan glycoprotein with proposed roles in peripheral nerve myelin formation. Duplication and deletion of the PMP22 gene is associated with Charcot-Marie-Tooth disease (CMT) and Hereditary Neuropathy with Pressure Palsies (HNPP) 15, 16, and several studies have shown that PMP22 regulates tumor development, metastasis, and invasion in different cancers in addition to its functions in neurodevelopmental and neurological disorders^17^-^28^, ^30^. However, the role of PMP22 in tumorigenesis and metastasis has been incompletely understood. In a previous study, PMP22 was found using the Explorer Antibody Microarray to be a cell surface protein(s) marker of self-renewing property and chemoresistance 31, 38. Bortezomib was tested in mice as a PMP22 inhibitor in combination with DDP for chemoresistant gastric cancer therapy, and the results showed improved tumor inhibition effect of combination therapy compared to the drug alone^31^. In our study, we also found anti-apoptotic properties of PMP22, and inhibition of PMP22 strongly suppressed tumor proliferation *in vitro* and *in vivo*. We used a chemotherapy drug, etoposide, to induce cell apoptosis, and found that PMP22 inhibited etoposide-induced cell apoptosis. Consistent with this finding, inhibition of PMP22 increased cell proliferation in gastric cells and the nude mice. Therefore, PMP22 inhibition may be a good target for treatment of gastric cancer.

In our results, we found that the effect of PMP22 overexpression on cell growth is not obvious (Supplemental Figures). It is possible that the expression of this protein itself is relatively high in tumor cells, however PMP22 inhibits etoposide-induced cell apoptosis after treatment of etoposide significantly. Knockdown of PMP22 inhibits cell growth and promotes the etoposide-induced proliferation suppression, and increases cell apoptosis in GC cells. Furthermore, PMP22 enhanced the inhibition of the p53 transcriptional activities and down-regulated the p53 targeting genes, including p21, BAX and PUMA with or without treatment of etoposide. In previous studies, PMP22 was reported to drive signal transduction away from the FAK/Src pathway and toward the AKT pathway, which reduced collagen gel contraction in ARPE-19 cells. Another study showed that Gas3/PMP22 expression was increased in apoptosis, that regulation of apoptosis by Gas3/PMP22 could regulate Schwann cell-differentiation^39^. Our results showed that PMP22 negatively regulated the p53 signaling pathway by inhibiting p53 target genes involved in apoptosis (BAX and PUMA) or cell cycle arrest (p21). However, the precise mechanism by which PMP22 regulates p53 is not clear. PMP22 is a tetraspan glycoprotein and p53 is locates on the cytoplasm and nucleus, therefore, they may not interact directly. The interactions of these two proteins require characterization.

## Supplementary Material

Supplementary figure.

## Figures and Tables

**Table 1:** The baseline characteristics of GC patients included (n = 40)

Clinicopathological parameters		PMP22-expression		P-value
	Case	low	high	
age, years				
>=50	22	9	13	0.5740
<50	18	3	15	
Gender				
Male	22	10	12	0.4852
Female	18	2	16	
Tumor size				
>=4	16	5	11	0.2587
<4	24	10	14	
TNM stage				
T1-T2	17	6	11	**0.0448**
T3-T4	23	8	17	
LN Metastasis				
T1-T2	15	4	11	**0.0482**
T3-T4	25	8	17	
		12	28	

P < 0.05 represents significant differences. Bold type indicates statistically significant difference.

**Table 2:** The detailed description of the GC patient tissues information (n = 40)

Number	Gender	Age	tumor size (diameter)	TNM stage	Pathological typing	Pathological typing
1	Female	74	≥4cm	Ⅲ	adenocarcinoma	Tubular adenocarcinoma
2	Male	45	<4cm	Ⅲ	adenocarcinoma	Low adhesion adenocarcinoma
3	Female	78	<4cm	Ⅱ	adenocarcinoma	Tubular adenocarcinoma
4	Male	39	≥4cm	Ⅱ	adenocarcinoma	Tubular adenocarcinoma
5	Female	68	<4cm	IV	adenocarcinoma	Low adhesion adenocarcinoma
6	Male	57	≥4cm	Ⅱ	adenocarcinoma	Tubular adenocarcinoma
7	Female	50	<4cm	IV	adenocarcinoma	mucosal adenocarcinoma
8	Male	58	<4cm	Ⅲ	adenocarcinoma	Tubular adenocarcinoma
9	Male	80	<4cm	IV	adenocarcinoma	Tubular adenocarcinoma
10	Male	57	<4cm	Ⅰ	adenocarcinoma	Tubular adenocarcinoma
11	Male	43	≥4 cm	IV	adenocarcinoma	Low adhesion adenocarcinoma
12	Male	76	<4cm	Ⅲ	adenocarcinoma	Low adhesion adenocarcinoma
13	Male	43	<4cm	Ⅲ	adenocarcinoma	Tubular adenocarcinoma
14	Male	66	<4cm	Ⅱ	adenocarcinoma	Low adhesion adenocarcinoma
15	Female	49	<4cm	Ⅲ	adenocarcinoma	Tubular adenocarcinoma
16	Female	49	<4cm	Ⅱ	adenocarcinoma	mucosal adenocarcinoma,
17	Female	82	≥4 cm	Ⅲ	adenocarcinoma	Tubular adenocarcinoma
18	Male	63	≥4 cm	Ⅱ	adenocarcinoma	Tubular adenocarcinoma
19	Female	73	≥4 cm	Ⅲ	adenocarcinoma	Papillary adenocarcinoma
20	Male	68	≥4 cm	Ⅱ	adenocarcinoma	Tubular adenocarcinoma
21	Female	47	≥4 cm	IV	adenocarcinoma	Tubular adenocarcinoma
22	Male	64	≥4 cm	Ⅱ	adenocarcinoma	Low adhesion adenocarcinoma
23	Male	49	≥4 cm	Ⅲ	adenocarcinoma	Tubular adenocarcinoma
24	Female	70	≥4 cm	Ⅲ	adenocarcinoma	Tubular adenocarcinoma
25	Male	46	≥4 cm	Ⅱ	adenocarcinoma	Tubular adenocarcinoma
26	Female	47	<4cm	Ⅲ	adenocarcinoma	Papillary adenocarcinoma
27	Male	60	<4cm	Ⅲ	adenocarcinoma	mucosal adenocarcinoma,
28	Female	62	≥4 cm	IV	adenocarcinoma	Papillary adenocarcinoma
29	Female	47	<4cm	Ⅱ	adenocarcinoma	Tubular adenocarcinoma
30	Female	43	≥4 cm	Ⅲ	adenocarcinoma	Tubular adenocarcinoma
31	Male	67	<4cm	Ⅱ	adenocarcinoma	Tubular adenocarcinoma
32	Male	59	<4cm	Ⅰ	adenocarcinoma	Tubular adenocarcinoma
33	Male	46	<4cm	Ⅲ	adenocarcinoma	mucosal adenocarcinoma,
34	Female	44	≥4 cm	Ⅱ	adenocarcinoma	Tubular adenocarcinoma
35	Male	57	<4cm	IV	adenocarcinoma	Tubular adenocarcinoma
36	Female	41	<4cm	Ⅲ	adenocarcinoma	mucosal adenocarcinoma,
37	Female	74	<4cm	Ⅱ	adenocarcinoma	Tubular adenocarcinoma
38	Male	48	<4cm	IV	adenocarcinoma	Low adhesion adenocarcinoma
39	Female	45	<4cm	Ⅱ	adenocarcinoma	Tubular adenocarcinoma
40	Male	44	<4cm	IV	adenocarcinoma	Low adhesion adenocarcinoma

## Abbreviations

GC: Gastric Cancer 1
PCD: programmed cell death
PMP22: Peripheral myelin protein 22
CMT: Charcot-Marie-Tooth disease
HGMEC: human glomerular microvascular endothelial cell line
OS: overall survival
NMI: N-Myc interactor
