# Supplementary material for: Inhibition of protein PMP22 enhances etoposide-induced cell apoptosis by p53 signaling pathway in Gastric Cancer
Source: Int J Biol Sci. 2021 Jul 25;17(12):3145–57. doi: 10.7150/ijbs.59825 (PMC8375224; doi:10.7150/ijbs.59825)
Supplement: Supplementary file 1 — Supplementary figure. [file ijbsv17p3145s1.pdf]

Inhibition of protein PMP22 enhances etoposide-induced cell apoptosis by p53 signaling pathway in Gastric Cancer

Jingjing Hou [1], Lin Wang[1], Jiabao Zhao[1], Huiqin Zhuo[1], Jia Cheng, Xin Chen, Wei Zheng, Zhijun Hong, Jianchun Cai*.

[a]Department of Gastrointestinal Surgery, Zhongshan Hospital of Xiamen University, Xiamen, Fujian 361004, China

[b]Institute of Gastrointestinal Oncology, Medical college of Xiamen University, Xiamen, Fujian 361004, China;

[c]Xiamen Municipal Key Laboratory of Gastrointestinal Oncology, Xiamen 361004, Fujian, China.

*Correspondence: caijianchun@xmu.edu.cn

Department of Gastrointestinal Surgery, Zhongshan Hospital, Xiamen University, Xiamen 361004, Fujian, China

[1]These authors contributed equally to the work.

Supplemental Figures

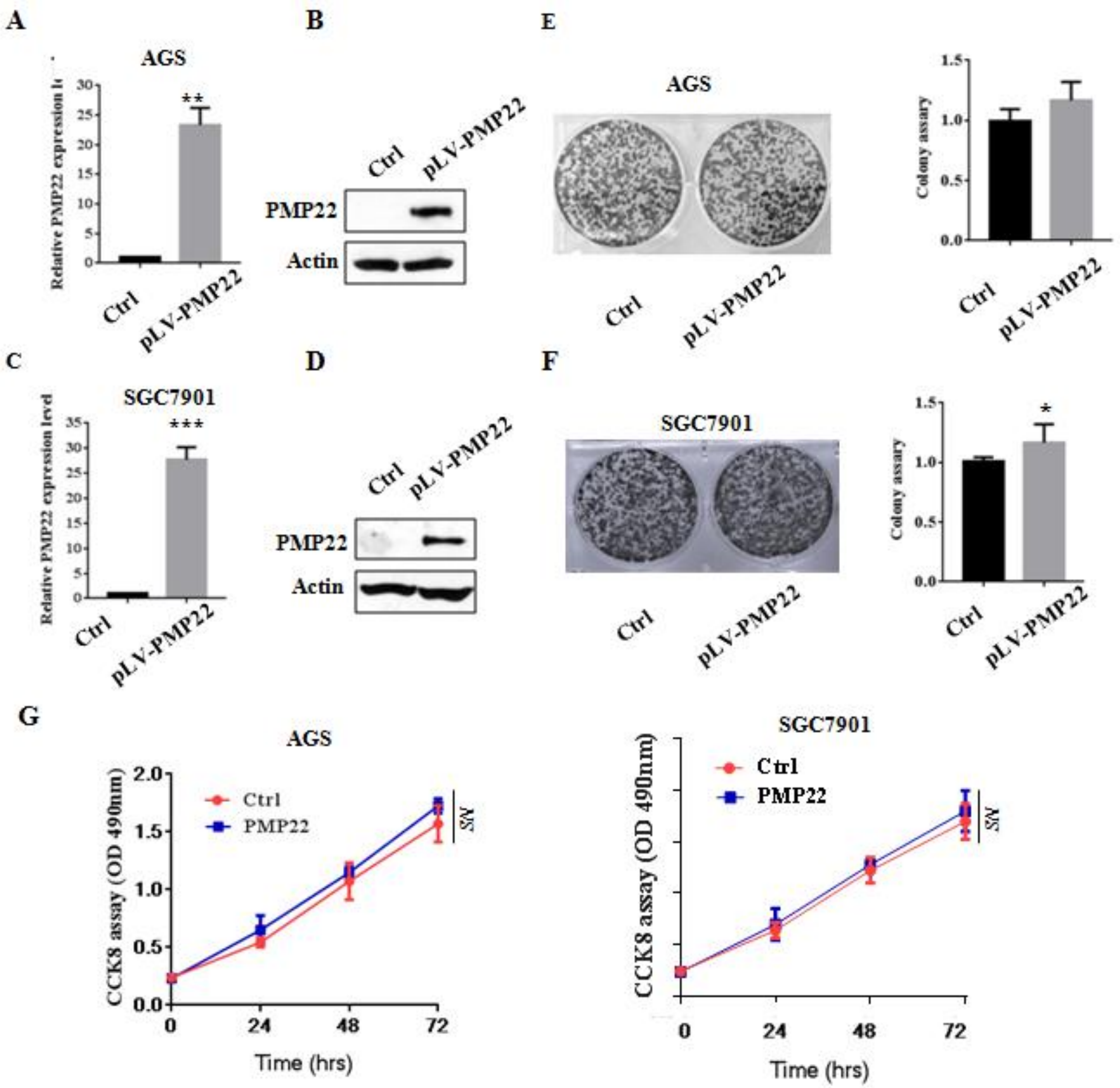

Supplemental Figure legends

Supplemental Figure. Overexpression of PMP22 has no effect on cell proliferation. (A-D) AGS and SGC7901 cells were infected with lentivirus expressing either pLKO-PMP22 or pLKO-Ctrl for 72 hours and then the mRNA and protein expression levels of PMP22 were examined by q-PCR (A,C) and Western blot (B,D). (E) Colony formation assay of AGS-Ctrl cells and AGS-PMP22 cells. (F) Colony formation assay of SGC7901-Ctrl cells and SGC7901-PMP22 cells. (G) Cell viability was determined by CCK-8 assay. Statistical analysis of the CCK-8 assay results at 72h was shown. Results are representative of three independent experiments, and the error bars represent the SD. *$p < 0.05$; ** $p < 0.01$; *** $p < 0.001$. NS, Not Significant.
